# Impact of ASUMA Intervention on HIV Risk Behaviors among Puerto Rican Adolescents

**DOI:** 10.3390/ijerph13010060

**Published:** 2015-12-22

**Authors:** Diana M. Fernandez-Santos, Christine Miranda-Diaz, Wanda I. Figueroa-Cosme, Raul O. Ramon, Angel M. Mayor, Eddy Rios-Olivares, Robert F. Hunter-Mellado

**Affiliations:** Internal Medicine Department, Universidad Central del Caribe, School of Medicine, PO BOX 60327, Bayamon 00960-6032, Puerto Rico; christine.miranda@uccaribe.edu (C.M.-D.); wanda.figueroa@uccaribe.edu (W.I.F.-C.); raul.ramon@uccaribe.edu (R.O.R.); amayorb@hotmail.com (A.M.M.); eddy.rios@uccaribe.edu (E.R.-O.); robert.hunter@uccaribe.edu (R.F.H.-M.)

**Keywords:** HIV prevention, adolescents, HIV risk factors, health disparities, Hispanics

## Abstract

The purpose of this manuscript is to assess and compare HIV risk behaviors among early adolescents after a three-year pilot study. A total of 135 public and private junior high schools students completed the intervention protocol. A self-administered questionnaire was given at baseline and at the end of the third year (fourth measure). Descriptive and inferential analyses were performed using SPSS 20.0. About 60% of the students were 14 years old at the fourth measure. The proportion of students that did not report at least one HIV risk behavior at baseline and those that reported any risk behavior at the fourth measure was lower in the intervention group (45.0%) than in the control group (54.5%). The proportion of students that reported at least one HIV risk behavior at baseline and those that did not report any HIV risk behavior at the fourth measure was higher in the intervention group than in the control group (33.3% *vs.* 8.3%). The proportion of students engaging in HIV risk behaviors was higher in the control group than in the intervention group at the fourth measure, suggesting that A Supportive Model for HIV Risk Reduction in Early Adolescence (ASUMA) intervention might be a promising initiative to reduce adolescents’ engagement in HIV risk behaviors.

## 1. Introduction

The target of the Joint United Nations Programme on HIV/AIDS (UNAIDS) is to get AIDS under control by 2030 [1]. According to UNAIDS, high-impact interventions should be developed to target individuals at high risk of HIV infection and to prevent new HIV infections among adolescents [1]. In Puerto Rico, the rate of HIV/AIDS diagnosis is highest among persons between 25 to 34 years old. By the end of 2012, about 700 new infections were reported in Puerto Rico, mostly due to intravenous drug use [2]. The Puerto Rico Consulta Juvenil biennial school survey indicated that alcohol was the most prevalent substance used among adolescents; 48.6% reported alcohol use during lifetime and 44.2% within the last year; followed by marihuana [3]. Papalia affirmed that tobacco, alcohol, and cannabis are the most prevalent drugs consumed by adolescents [4]. These substances have the potential to predispose adolescents to engage in high risk behaviors [4]. About 30.8% of the students reported having sexual intercourse and 51.5% had their first sexual encounter before the age of 15 [3]. High school students were more sexually active (43.7% *vs.* 19.6%) than junior students [3]. In the United States, hispanic adolescent males below the age of 15 engage more frequently in sexual activity than girls [5].

High transmission rates of sexually transmitted infections (STI) in adolescents have been related to lack of HIV knowledge, low self-esteem, invulnerability feelings toward STI/HIV, and higher sensation seeking characteristics [5]. Peer pressure has a negative influence during adolescence that often results in experimentation with tobacco, alcohol, and illegal drugs [6]. Also, peer pressure might affect the early onset and prevalence of sexual behaviors [6]. Researchers have stated that adolescents are strongly influenced by their peers than by older adults [7,8]. Another factor, commonly observed during adolescence is sensation seeking. Researchers have studied the relationship between risky behaviors and sensation seeking among adolescents. Sensation seeking has been proven to mediate the relationship between pubertal development and drug use among adolescents [9]. High alcohol consumption in students was directly associated with higher sensation seeking [10]. Pound (2015) indicated that adolescents with a family history of alcohol and drug use have an increased predisposition for developing substance use disorders [11].

Parental support is crucial in the implementation of HIV prevention strategies among adolescents. Many programs do not include parents in their HIV youth prevention efforts because parental involvement is often difficult [12]. Open discussion with parents can help delay sexual debut, protect adolescents from engaging in risky behaviors, and support the healthy adolescent’s sexual socialization [13]. Moreover, adolescents who perceive positive family support, family closeness, parental monitoring, and parent-adolescent communication are less likely to engage in risky sexual behavior and substance use [14,15].

The purpose of this manuscript is to assess and compare the HIV risk behaviors among intervention and control groups after a three year pilot study. The objectives are to: (1) describe the HIV risk behaviors profile of Puerto Rican early adolescents; and (2) analyze differences in HIV risk behaviors among study groups.

## 2. Experimental Section

This pilot study is a prospective cohort study in which students in the intervention group received a school-based intervention known as A Supportive Model for HIV Risk Reduction in Early Adolescence (ASUMA). The students in the control group only received HIV/AIDS educational materials.

### 2.1. Theoretical Framework

**Figure 1:**
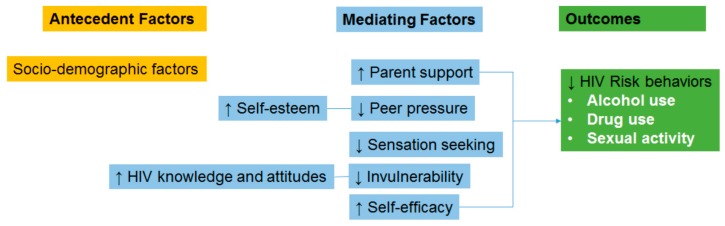
A Supportive Model for HIV Risk Reduction in Early Adolescence (ASUMA) theoretical framework.

We developed a theoretical framework built on existing literature to explain the impact of developmental factors and parental support, as a mean to reduce HIV risk behaviors in early adolescents (*i.e.*, alcohol use, drug use, and sexual activity). The theoretical framework components and the ASUMA curriculum have been published elsewhere [7,16,17]. The ASUMA intervention consists of eight workshops that were implemented during three academic years: four workshops in year 1 (seventh grade), two workshops in year 2 (eighth grade), and two workshops in year 3 (ninth grade). The intervention is directed to increase the adolescent’s self-efficacy, self-esteem, and HIV/AIDS knowledge and attitudes, while decreasing negative peer pressure, sensation seeking, and invulnerability (See Figure 1). Each workshop was delivered using small group discussion, role play, debates, brainstorming, patient testimony, and critical thinking as educational strategies. ASUMA intervention includes parental support as a core element. Parents from the intervention group were invited to participate in a four-hour parent’s workshop. We performed a culturally-informed adaptation of an evidence-based intervention known as “Talking with Kids about AIDS: A Program for Parents and Other Adults Who Care” developed by the HIV/AIDS Education Project at Cornell University. Thirty-five (53%) parents participated in the workshop.

### 2.2. Subjects and Data Collection

This pilot study was approved by the Universidad Central del Caribe Institutional Review Board (IRB) and was conducted between August 2004 and May 2009. We selected a purposive sample of four junior high schools from the Puerto Rico metropolitan area. Each school was randomly assigned to the intervention or control group (one public school and one private school in each group). Students were recruited in seventh grade and were followed until ninth grade after an assent and an informed consent were discussed and signed. At baseline (seventh grade), 173 early adolescents were recruited. We analyzed changes in HIV risk behaviors of students that completed the three year period (*n* = 135 students). Sixty-six students from the intervention group and 69 students from the control group completed the ASUMA project. School transfers were the main reason for attrition.

### 2.3. Measures

A previously validated self-administered questionnaire was given at baseline (seventh grade) and at the end of each academic year. The research questionnaire was validated in terms of face validity, content validity, and construct validity, as described elsewhere [16,17]. The questionnaire and curriculum were approved by the Institutional Review Board at the Universidad Central del Caribe, School of Medicine. Study variables included demographic factors and HIV risk behaviors. The HIV risk behaviors variable was defined as reporting at least one of the following behaviors: sexual intercourse, sexual intercourse without condom (for those who reported having sexual intercourse), cannabis use, and alcohol use. For the purpose of this manuscript, baseline measure (seventh grade) and the fourth measure (ninth grade) reported behaviors were analyzed.

### 2.4. Data Analysis and Certification of Methods

Descriptive and inferential analyses were conducted. The McNemar paired test was performed to analyze differences in proportions among matched variables in pre and post HIV risk behaviors by each study group. The overall significance level was set to 0.05. *IBM SPSS Statistics for Windows*; Version 20.0 (IBM Corp., Armonk, NY, USA, 2011) was used to perform the statistical analyses. The research staff of the Data Management and Statistical Research Support Unit (DMSRSU) performed the statistical analysis.

## 3. Results and Discussion

### 3.1. Results

A total of 135 public and private junior high schools students completed the ASUMA project (49% intervention *vs.* 51% controls). At the end of the pilot study period (ninth grade), 61% of the sample were 14 years old and the next most prevalent group were 15-year old students (28%). Table 1 shows sociodemographic and HIV risk behaviors profile at the fourth measure by the study group. Most students in the intervention group were male (58%) and 47% of students reported at least one HIV risk behavior in the last year. Most students in the control group were female (62%) and 61% of students reported at least one HIV risk behavior in the last year. Most students reported alcohol use (48% *vs.* 60%); followed by sexual intercourse (8% *vs.* 21%) and cannabis (2% *vs.* 4%) in the intervention group and control group, respectively.

**Table 1:** Sociodemographic and HIV risk behaviors profile at the end of the study.

Variables	Intervention Group (*n =* 66)		Control Group (*n =* 69)	
	n	%	n	%
**Gender**				
Male	38	57.6	26	37.7
Female	28	42.4	43	62.3
**Cannabis use**				
Yes	1	1.6	3	4.5
No	62	98.4	63	95.5
**Alcohol use**				
Yes	31	48.4	40	59.7
No	33	51.6	27	40.3
**Sexual intercourse**				
Yes	5	8.1	14	20.6
No	57	91.9	54	79.4
**Sex without condom**				
Yes	0	0	12	85.7
No	5	100	2	14.3
HIV risk behavior *				
Yes	31	47.0	41	61.2
No	35	53.0	26	38.8

Note: * Reporting at least one of the following HIV risk behaviors: sexual intercourse, sexual intercourse without condom, cannabis use and/or alcohol use.

**Table 2:** Comparison among HIV risk behaviors: Baseline *vs.* fourth follow-up measure.

Baseline Measure	Fourth Follow-Up Measure			
	Intervention Group		Control Group	
	Yes n (%)	No n (%)	Yes n (%)	No n (%)
HIV risk behavior *^,^**				
Yes	4 (66.7)	2 (33.3)	11 (91.7)	1 (8.3)
No	27 (45.0)	33 (55.0)	30 (54.5)	25 (45.5)
Alcohol use *				
Yes	4 (80.0)	1 (20.0)	11 (91.7)	1 (8.3)
No	27 (45.8)	32 (54.2)	29 (52.7)	26 (47.3)
**Cannabis use**				
Yes	0 (0)	0 (0)	0 (0)	0 (0)
No	1 (1.2)	55 (98.2)	3 (5.0)	57 (95.0)
**Sexual intercourse**				
Yes	0 (0)	0 (0)	0 (0)	0 (0)
No	3 (33.3)	6 (66.7)	12 (85.7)	2 (14.3)
**Sexual intercourse without condom**				
Yes	0 (0)	0 (0)	0 (0)	0 (0)
No	0 (0)	5 (100)	2 (14.3)	12 (85.7)

Notes: McNemar’s test, * *p* < 0.001; ** Reporting at least one of the following HIV risk behaviors: sexual intercourse, sexual intercourse without condom, cannabis use and/or alcohol use.

Table 2 shows changes in HIV risk behaviors at baseline and fourth follow-up measures by the study group. The proportion of participants who did not report at least one HIV risk behavior at baseline but reported any HIV risk behaviors at the fourth measure was lower in the intervention group (45.0%) than in the control group (54.5%). The same trend was observed after analyzing each risk behavior individually: alcohol use (45.8% *vs.* 52.7%); cannabis use (1.2% *vs.* 5.0%); sexual intercourse (33.3% *vs.* 85.7%) and sex intercourse without condom (0.0% *vs.* 14.3%). The proportion of students who reported at least one HIV risk behavior at baseline but did not report any HIV risk behaviors at the fourth measure, was higher in the intervention group than in the control group (33.3% *vs.* 8.3%). The same trend was observed after analyzing alcohol use separately (20.0% *vs.* 8.3%). Significant differences among proportions were observed among students who reported alcohol use in the last year and in the constructed variable HIV risk behavior (McNemar Chi-square, *p* < 0.001).

### 3.2. Discussion

This pilot study compared HIV risk behaviors in a sample of students that were followed for three academic years. ASUMA is a theory-based prevention project aimed at explaining why early Puerto Rican adolescents engage in HIV risk behaviors. A higher proportion of students in the control group reported alcohol use, sexual intercourse, and cannabis use in the last year than in the intervention group. During adolescence, experimentation with tobacco, alcohol, illegal drugs, and early onset of sexual intercourse has been linked with negative peer pressure [8,9]. The Adolescents’ engagement in HIV risk behaviors might be explained by the increased sensation-seeking attitude that is often observed during this developmental period [10]. ASUMA’s previous findings showed a significant increase in HIV/AIDS knowledge among students in the intervention group (Mdn *=* 19 *vs.* Mdn *=* 22; *p < 0.*001) whereas no changes were observed in the control group [17]. Positive improvements in the developmental factors at the end of the implementation phase in the intervention group were also observed [17].

At the end of the pilot study, we found a lower proportion of students reporting at least one HIV risk behavior in the intervention group than in the control group. This finding suggests promising evidence that ASUMA reduces adolescent’s engagement in HIV risk behaviors. To be effective, HIV prevention programs targeting adolescents should address developmental and HIV risk-related factors, and integrate parents as part of the core elements, especially among the Hispanic population [16,17]. ASUMA-tailored intervention is focused on increasing parental support, self-efficacy, self-esteem, HIV/AIDS knowledge and attitudes toward HIV/AIDS, while decreasing the sense of peer pressure, sensation seeking, invulnerability, and peer pressure. A study limitation is that the sample is not probabilistic; however, it has representation from public and private school systems in Puerto Rico. In terms of attrition proportion (135/173; 22%), we understand that a different HIV risk behaviors profile might be obtained if all students completed the pilot study. Also, the sample size did not allow for the performance of a multivariate analysis.

## 4. Conclusions

Adolescence is a stage of experimentation and exploration. This developmental period is associated with risk-taking practices. Tailored interventions should be developed to reduce future HIV infections, which are often experienced in minority communities. HIV prevention interventions targeting adolescents should include: pragmatic strategies, cultural aspects, and the development of coping skills to facilitate the process of active learning [7]. The role of parents in HIV prevention has been reported elsewhere. Parents, especially in the Hispanic community, play an important role in the delay of sexual intercourse and drug use through effective communication and trust development. ASUMA’s theoretical framework could be adapted to other minority populations as a means to reduce HIV risk behaviors in adolescent populations.

## Supplementary Files

Supplementary File 1
