# Supplementary material for: Impact of ASUMA Intervention on HIV Risk Behaviors among Puerto Rican Adolescents
Source: Int J Environ Res Public Health. 2015 Dec 22;13(1):60. doi: 10.3390/ijerph13010060 (PMC4730451; doi:10.3390/ijerph13010060)
Supplement: Supplementary File 1 [file ijerph-13-00060-s001.pdf]

International Journal of
Environmental Research and Public Health

MDPI

Supplementary Materials: ASUMA Adolescent Curriculum

Table S1. Curriculum of the study: a supportive model for HIV risk reduction in early adolescent (ASUMA project).

Title of Curriculum Unit	Objectives	Strategies	Evaluation
Unit 1—HIV/AIDS attitude knowledge and vulnerability	• Participants will increase their HIV/AIDS knowledge.<br>• Participants will decrease their invulnerability to HIV/AIDS.	• Clue activity<br>• Peer group discussion<br>• Brainstorm<br>• Audiovisual presentation<br>• Debate<br>• Critical thinking<br>• Round-Robin<br>• Patient testimony<br>• Individual reflection	• Pre and Post-test and workshop evaluation.<br>• Evaluation of outcome activities.<br>• Follow-up questionnaire.
Unit 2—HIV/AIDS risk	• Participants will increase their resilience to HIV/AIDS risk behaviors.<br>• Participants will decrease their invulnerability to HIV/AIDS risk vulnerability.	• Audiovisual presentation<br>• Individual critical thinking<br>• Peer discussion<br>• Critical discussion<br>• Group discussion<br>• Round-Robin	• Pre and Post-test and workshop evaluation.<br>• Evaluation of outcome activities.<br>• Self-administered questionnaire.
Unit 3—Self-esteem: "I'm important"	• Participant will increase their self-esteem and self-efficacy.	• Individual reflection<br>• Dramatization<br>• Peer discussion<br>• Group discussion<br>• Role-Play<br>• Critical thinking<br>• Bowl-Fish<br>• Critical discussion	• Pre and Post-test and workshop evaluation.<br>• Evaluation of outcome activities.<br>• Self-administered questionnaire.
Unit 4—Peer pressure: "Because I'm value person"	• Participants will describe how peer pressure could have a negative influence on them.<br>• Participants will develop a plan to resist the negative peer pressure.	• Individual reflection<br>• Visual presentation<br>• Critical thinking<br>• Discussion group<br>• Role-Play<br>• Group discussion	• Pre and Post-test and workshop evaluation.<br>• Evaluation of outcome activities.

Table S1. *Cont.*

Title of Curriculum Unit	Objectives	Strategies	Evaluation
Unit 5—Booster: "I can prevent risky situations"	• Participants will develop skills on how to manage sensation seeking.	• Critical thinking<br>• Group discussion<br>• Spider web<br>• Brainstorm<br>• Discussion group<br>• Cardboard	• Pre and Post-test and workshop evaluation.<br>• Evaluation of outcome activities.
Unit 6—"I can perceive the media negative influence and prevent HIV risky behavior if…"	• Participants will discriminate the media influence.<br>• Participants will develop skills to manage media influence and prevent risky situations.	• Critical thinking<br>• Discussion group<br>• Collage<br>• Peer group discussion<br>• Brainstorm<br>• Situation	• Pre and Post-test and workshop evaluation.<br>• Evaluation of outcome activities.
Unit 7—"I'm able to resist HIV risk behavior"	• Participants will increase participants' self-efficacy skills to avoid HIV risks behaviors.	• Game<br>• Peer Group Discussion<br>• Individual Critical thinking<br>• Audiovisual	• Pre and Post-test and workshop evaluation.<br>• Self-administered questionnaire.
Unit 8—"I'm ready for my future"	• Participants will demonstrate self-efficacy skills to avoid HIV risks behaviors.	• Role Play<br>• Drama<br>• Song Story<br>• Short film<br>• Peer Group Discussion<br>• Dynamic: Mail to the future<br>• Individual Critical Thinking	• Pre and Post-test and workshop evaluation.<br>• Evaluation of outcome activities.

© 2015 by the authors; licensee MDPI, Basel, Switzerland. This article is an open access article distributed under the terms and conditions of the Creative Commons by Attribution (CC-BY) license (http://creativecommons.org/licenses/by/4.0/).
